# Clostridium Bacteria and Autism Spectrum Conditions: A Systematic Review and Hypothetical Contribution of Environmental Glyphosate Levels

**DOI:** 10.3390/medsci6020029

**Published:** 2018-04-04

**Authors:** Isadora Argou-Cardozo, Fares Zeidán-Chuliá

**Affiliations:** 1 Specialization Course in Public Management and Regional Development, Faculty of Administration and Tourism, Federal University of Pelotas (UFPel), 96010-610 Pelotas, RS, Brazil; argou2@gmail.com; 2 Departamento de Ciencias Biomédicas Básicas, Facultad de Ciencias Biomédicas y de la Salud, Universidad Europea de Madrid, 28670 Villaviciosa de Odón, Spain; 3 Faculty of Medicine, University of Turku, FI-20520 Turku, Finland

**Keywords:** Clostridium, bacteria, gut microbes, gene-environment interactions, pollution, pesticides, agrotoxics, brain, autism spectrum disorders

## Abstract

Nowadays, there seems to be a consensus about the multifactorial nature of autism spectrum disorders (ASD). The literature provides hypotheses dealing with numerous environmental factors and genes accounting for the apparently higher prevalence of this condition. Researchers have shown evidence regarding the impact of gut bacteria on neurological outcomes, altering behavior and potentially affecting the onset and/or severity of psychiatric disorders. Pesticides and agrotoxics are also included among this long list of ASD-related environmental stressors. Of note, ingestion of glyphosate (GLY), a broad-spectrum systemic herbicide, can reduce beneficial bacteria in the gastrointestinal tract microbiota without exerting any effects on the Clostridium population, which is highly resistant to this herbicide. In the present study, (i) we performed a systematic review to evaluate the relationship between Clostridium bacteria and the probability of developing and/or aggravating autism among children. For that purpose, electronic searches were performed on *Medline/PubMed* and *Scielo* databases for identification of relevant studies published in English up to December 2017. Two independent researches selected the studies and analyzed the data. The results of the present systematic review demonstrate an interrelation between Clostridium bacteria colonization of the intestinal tract and autism. Finally, (ii) we also hypothesize about how environmental GLY levels may deleteriously influence the gut–brain axis by boosting the growth of Clostridium bacteria in autistic toddlers.

## 1. Introduction

Autism and autism spectrum disorders (ASD) are indistinctively used terms to define a group of disorders of brain development. ASD include autistic disorder, Asperger’s syndrome, pervasive developmental disorder not otherwise specified (PDD-NOS), and childhood disintegrative disorder [1]. For decades, the etiology of ASD has been strongly controversial and numerous researchers have proposed their own so-called “autistic gene” and innumerous environmental stressors [2,3,4] to explain the apparent rapid increase in incidence rates over the last 20 years, with 4:1 male to female ratio prevalence [5]. Overall, the causes of such differences are not completely understood. 

Our group has studied ASD from a multifactorial perspective and recently proposed a consensus in silico network model “GENVI” capable of integrating reported and other novel putative gene–environment interactions in autism. The network interconnected 122 genes/proteins and 191 factors (drugs, pollutants, chemicals and pesticides and other stressors) through 1461 interactions [6]. In this network model, the Ras-related C3 botulinum toxin substrate 1 (Rho GTPase RAC1) displayed a central topological position at the interface between genetic and environmental factors of ASD and, in general, the Rho family of GTPases showed differential expression in the cerebellum of autistic patients when compared to control samples [6,7]. 

Appropriate early-life assembly of the gut microbiota is believed to play a critical role in subsequent emotional and cognitive development. The brain-gut-microbiota axis is defined as a communication network between the brain, the gut, and the bacteria residing there. Both development and maintenance of a diverse gut flora is crucial for accomplishing successful behavioral tasks and proper physiology and structure of the brain [8]. In 2017, we postulated that the differential expression of the Rho family of small GTPases found in the cerebellum of patients [6,7] would be consistent with recent reports supporting Clostridium spores as key elements in the etiology of autism [9], since the Ras and Rho family of GTPases are specifically targeted by clostridial toxins [10]. Moreover, while preparing this manuscript an elegant case study [11] reported elevated urinary glyphosate (GLY) and Clostridium-related metabolites with altered dopamine metabolism in triplets with ASD or suspected seizure disorder. Thus, in the present systematic review, we search for literature evidence regarding the interrelation between the presence of Clostridium bacteria and the etiology of autism and thereafter, we postulate about how the environmental exposure to GLY, post-emergent systemic herbicide with activity on essentially all annual and perennial plants, may potentially contribute to the disruption of the gut-brain axis by facilitating the growth of clostridial species.

## 2. Materials and Methods 

### 2.1. Systematic Review Question

Is there an interrelation between clostridium bacteria colonization of the intestinal tract and the neurological symptoms associated with ASD patients?

### 2.2. Search Strategy

The literature was screened to identify published articles linking the colonization of Clostridium bacteria of the intestinal tract and autism. Comprehensive and systematic electronic searches were performed on *Medline/Pubmed* (https://www.ncbi.nlm.nih.gov/pubmed/) and *Scielo* (http://www.scielo.org/php/index.php?lang=es) Databases by two independent investigators (IAC and FZC), started from 8 January 2017 until 30 November 2017. The following terms were used: (tw: [“Clostridium” or “Clostridia” or “Clostridial species” or “Clostridial spores”]) AND (tw: [Autism] or [Autism spectrum disorders] or [Autistic patients] or [ASD]. Duplicate references were excluded and analyzed by the same two reviewers (I.A-C. and F.Z-C.) based on inclusion and exclusion criteria. In addition to the electronic search, the authors performed a hand search of the reference lists from the selected articles, for identifying potentially missing studies that may deserve analysis in the present systematic review.

### 2.3. Selection Criteria

Studies were included only if they dealt with evidence linking the colonization of Clostridium bacteria with neurological symptoms and/or etiology of ASD on human individuals, without time restrictions, based on the following inclusion criteria:

- Only studies published in English.
- Clinical studies in humans.
- Neurological symptoms and/or diagnosed autism must be present.
- Studies that allow access to their specific data.
- In case of duplicate publications, only the most recent data will be preferred.

### 2.4. Selection of Studies

The detailed screening was independently conducted by two reviewers (I.A-C. and F.Z-C.) with the predefined eligibility criteria. All articles were identified and classified by their title and abstract to selection criteria. After previous screening of titles and abstract, the full articles were evaluated by the same reviewers. The cases of disagreement between authors were solved after discussion. For the next screening phase, the complete scientific publication was read and analyzed. Data regarding the study design, sample size, study characteristics, year of publication, and limitations were collected from each included study. 

## 3. Results

**Figure 1:**
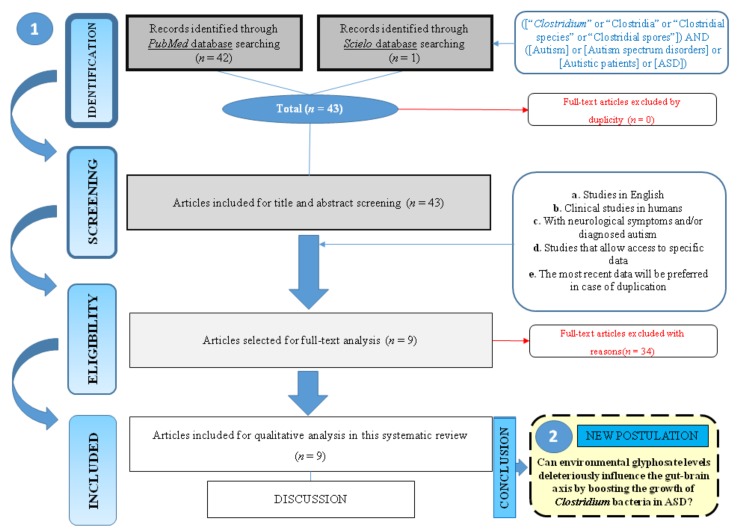
Graphical abstract representing (1) the article selection flow chart of the systematic review and (2) final postulation.

The first electronic search resulted in 42 titles from *Medline/Pubmed* and one from *Scielo* databases. We found no duplicate papers in this first analysis. Thus, a total of 43 titles were considered for potential inclusion. Of these, 34 articles were removed based on title and abstract (theoretical reviews and hypothesis were not considered), resulting in nine full texts that were identified, according to the inclusion criteria in this systematic review. Figure 1 displays the flowchart of the study selection. The samples in the included studies totaled 495 individuals (310 patients and 185 controls). Regarding the type of samples, seven studies utilized human stools, one analyzed urine samples and one investigated intestinal biopsies (Table 1).

Finegold et al. [12] in 2002 performed a series of microbiological studies of the intestinal content of autistic individuals and compared their results to those obtained in control children, with the hope of finding a unique and characteristic flora to the disorder. To the best of our knowledge, this report was the first demonstrating the existence of nine clostridial species in fecal samples from children with autism compared with only three found in control children. Non-clostridial anaerobes and microaerophilic bacteria were common in upper gastrointestinal specimens of autistic children but were not detected in healthy subjects. Perhaps, the main limitation of the study resides on its methodology of culture-based methods, which sometimes lead to significant underestimation of bacteria present in fecal samples. Since the authors were aware about the need to improve the sensibility of their approach, two years later, Song and collaborators (same research group) [13] studied the composition of intestinal flora based on the detection of rRNA genes (Real-Time PCR Quantitation, RT-PCR). Their analysis by RT-PCR showed that cell count differences between autistic children and healthy subjects for *Clostridium bolteae* and the following Clostridium groups were statistically significant. Nevertheless, the authors indicated the need for further confirmations in large numbers of samples. Since numerical differences in the gut flora of ASD patients and healthy subjects needed further research, in 2005, Parracho et al. [14] performed an analysis of fecal samples from 58 children with autism by fluorescent in situ hybridization (FISH) using group-specific oligonucleotide probes and compare it with two healthy control groups (a non-autistic sibling group and an unrelated healthy group. Their data reported a higher incidence of the toxin producer *Clostridium histolyticum* group (Clostridium clusters I and II) when compared to healthy children. However, these results differed for the second control group (non-autistic sibling) where intermediate level of the *C. histolyticum* group was observed with no significant differences from any other analyzed groups. These results not only support the hypothesis of a link between clostridia and the development of certain autistic features but also suggest a putative therapeutic approach of autistic symptoms based on the modulation of gut microflora by reducing the numbers of Clostridium bacteria in ASD patients, while stimulating more beneficial gut bacteria (e.g., Lactobacillus or Bifidubacterium). Nonspecific symptoms are often related with intestinal inflammation and elevated level of fecal lactoferrin (FLA) resistant to proteolysis and bacteria degradation [15]. Martirosian et al. successfully found higher levels of FLA in stools from autistic children when matched with healthy controls [16] and even though toxins were not detected, *C. perfringens* were isolated significantly often from the autistic samples. In particular, intermediate sensitive strains to penicillin 19%, to clindamycin 11.3%, and to metronidazole 7.5%. Unfortunately, the studied groups were not big enough and additional confirmation of these data with larger groups was recommendable and the elucidation potential mechanism contributing to the negative consequences associated with ASD by studying the role of the gut microbiome and metabolome, considering that metabolome refers to the complete set of small-molecule chemical found within a biological sample if diverse origins. De Angelis et al. (2013) followed this approach [17] and compared total and active fecal microbiota from autism, PDD-NOS patients, and healthy controls through a culture-dependent and -independent methods as well as metabolomic analyses. Caloramator, Sarcina and Clostridium genera were the highest in autistic children. Compared to healthy individuals, the composition of Lachnospiraceae family was also especially different in the autistic group and PDD-NOS. In addition, phenol compounds such as phenol, 4-(1,1-dimethylethyl)-phenol, p-cresol) and free amino acids (Asp, Ser, Glu, Gly, Ala, Val, Ile, Phe, His, Tpr, Lys and Pro) were elevated in the feces of PDD-NOS and even more in samples from autistic subjects. These results are therefore consistent with the previous reports [12,13,14,15,16], suggesting that certain microbial and metabolic profiling could represent signatures for ASD, and invites for further investigation regarding additional markers and the pathophysiological roles of intestinal dysbiosis in these children. Xiong et al. [18] proposed a gas chromatography/mass spectrometry-based urinalysis to identify the metabolomic profile of these patients and demonstrated higher (*p* < 0.001) concentrations 3-(3-hydroxyphenyl)-3-hydroxypropionic acid (HPHPA), 3-hydroxyphenylacetic acid (3HPA), and 3-hydroxyhippuric acid (3HHA) are present in these subjects when compared to healthy controls. Such values significantly decreased once these patients were submitted to vancomycin treatment indicating their Clostridium species-derived origin and suggesting these markers as putative predictors of ASD. In general, intestinal dysbiosis could be related to (i) yeast infection or (ii) leaky gut. One year later, Iovene and collaborators [19] searched for evidence demonstrating the presence of gastrointestinal *Candida albicans* finding higher counts in autistic samples by using a diagnostic cultural approach. Nevertheless, it is unclear whether pharmacological-based therapies to eliminate yeasts in this patient could be useful to improve the behavioral hallmarks of the disorder. 

In general, the differences in the intestinal bacteria of autistic toddlers were well demonstrated when compared with healthy individuals. However, it was poorly characterized whether those differences could be specific for an intestinal area or generalized in the whole intestine. In contrast to the fecal and ileal microbiome, the bacterial profile in the duodenum of ASD patients required more extensive investigation. This study was performed by Kushak et al. (2017) [20] and reported statistically significant differences in duodenal microbiota at the genus and species levels between children with ASD and controls, together with changes in the taxa associated with disaccharidase activity. Nevertheless, the studied group showed heterogeneity in age and sex and as well as absence of one control group without autism and gastrointestinal symptoms to compare with, representing a limitation of this study. That said, Finegold´s group [21] decided to closely explore the potential role of clostridial species in ASD by isolating *C. perfringens* strains to perform PCR analysis for the main *C. perfringens* toxin genes, α, β, β2, ε, ι and *C. perfringens* enterotoxin gene. Their results showed significantly higher counts of *C. perfringens* (*p* = 0.031) when compared to control samples, but also suggested that the involvement of β2-toxin in autistic patients with gastrointestinal disease is significant, with 79% of the 33 autism patients showing the presence of β2-toxin gene in feces, compared to 38% of the 13 control subjects (*p* = 0.014). 

All these evidences together support that dysbiosis of the gut microbiota, in general, and the proliferation of intestinal clostridia may contribute to the clinical picture of ASD, likely functioning as key elements in the etiology of autism.

## 4. Discussion: An Interrelation between Glyphosate, Clostridium Bacteria, and Autism?

Nowadays, several researchers are paying attention to “gut dysbiosis” or a state of imbalance in the gut microbial ecosystem, which includes excessive proliferation of specific organisms and loss of others as a potential cause for several diseases such as cancer, obesity, and Alzheimer´s disease [22,23,24]. To the best of our knowledge, first observations were made by Sudo and colleagues in 2004 in experimental mice. The authors observed that the lack of normal gut microbiota could have a significant impact on adult stress responsiveness and that such alterations could be partially reversed by its re-colonization [25]. It has been postulated that vaginal microflora likely contributes to the initial seeding of the neonatal microbiome during vaginal deliveries, but evidence highlighting that the uterine environment may not be sterile, suggests that colonization newborns may occur even before birth, with a potential total contribution of microbiota from maternal oral, vaginal, urinary tract, and intestine for these colonizing bacteria [26]. The theories regarding the inter-relation between gut microbes and the brain are diverse, from concepts postulating that humans are just the vehicle for the 100 trillion microorganisms living inside of us, to our gut microbiota may have developed ways to “hack” into our reward system, in order to make us look for certain foods and avoid others that are most beneficial to them [27]; or even a putative role of the host-microbe interactions in mammalian brain for development and evolution. Despite the intense research, this mutual interplay has largely been neglected by clinicians working in neurology and psychiatric areas. With respect to disorders of the central nervous system (CNS) and psychiatric conditions, an overgrowing number of reports are linking the disruption of microbial diversity with depression and anxiety, irritable bowel syndrome (IBS), attention-deficit hypersensitivity disorder (ADHD), and ASD [28,29]; being this last one the focus of our systematic review.

Finegold and collaborators identified a number of circumstantial reasons to consider gut microbiota as a potentially etiological factor involved in late-onset autism including: (i) the onset of the disease is often followed by antimicrobial therapy, (ii) intestinal symptomatology are common in these patients, (iii) the administration of antibiotics (e.g., oral vancomycin) may lead to certain improvements but a relapse may occur, on the other hand, when vancomycin administration is interrupted [12]. Pathogenicity of several anaerobic bacteria and their virulence is based on secreted toxins, mainly produced by clostridial species. Despite being non-invasive bacteria, these microbes can exert long-distance harmful effects (e.g., brain) if their secreted molecules pass through the intestinal barrier and disseminate by the general circulation to remote organs or tissues, where they are active [30]. For instance, it is known that *Clostridium difficile* toxin B can trigger programmed cell death in granule neurons and changes in density and spine morphology [31]. We started paying attention to Clostridium bacteria in the context of autism once we pointed towards the Rho family of small GTPases, by using in silico-based and systems biology approaches, as a potential minimum common denominator in ASD at the molecular interface between genetic and environmental factors, and confirmed its aberrant differential gene expression in the cerebellum of patients when compared to control subjects [6,7]. Bakos´group is actively following a similar direction, studying mechanisms underlying neuritogenesis, axon pathfinding and the role of GTPases in neurite outgrowth, with special emphasis on alterations associated with ASD [32,33]. 

On the other hand, the etiological association between agricultural pesticides and ASD is not recent [34,35,36,37,38]. One can find evidence for an increased risk of ASD diagnosis among children whose mothers lived during pregnancy close to fields where pesticides were applied. Specifically, the authors found a significant link between ASD and application of non-specified organophosphates during the third trimester as well as one specific organophosphate, chlorpyrifos, during the second trimester of pregnancy [38].

GLY or *N*-(phosphonomethyl)glycine is a broad-spectrum phosphate herbicide. GLY is the active ingredient in the herbicide Roundup, which is the most heavily used herbicide worldwide. Only in the United States, around 300 million pounds are applied each year [39,40,41]. This raises the question whether GLY may have a potential role, similar to chlorpyrifos, on ASD prevalence. Coming back to the interplay between Clostridium species and the probability of developing and/or aggravating autism among children, environmental GLY levels may deleteriously influence the gut–brain axis. Highly pathogenic bacteria such as *Clostridium perfringens* and *Clostridium botulinum* among others are known to be highly resistant to GLY [42]. On the contrary, most of beneficial bacteria (e.g., *Enterococcus faecalis*, *Enterococcus faecium*, *Bacillus badius*, *Bifidobacterium adolescentis* and *Lactobacillus* spp.) were shown to be moderate to highly susceptible. The normal intestinal microflora is a critical for preventing intestinal colonization by *Clostridum botulinum*, as it has been shown in the mouse model of infant botulism. In this sense, the preferential GLY toxicity to the most prevalent *Enterococcus* spp. in the gastrointestinal tract could be, at least in theory, a significant predisposing factor that is associated with the increase in *C. botulinum* [43].

Moreover, another additional study by Ackerman group in 2015 [44] added further evidence confirming this postulation, showing results of a GLY-induced inhibitory effect on specific groups of ruminal microbiota but increased the population of pathogenic species. In humans, we can find a recent interesting case report where *Clostridium tertium* was identified in a 44-year-old female who attempted suicide by GLY ingestion [45] and You et al. further hypothesized that GLY may be a predisposing factor responsible for the pathogenesis of these bacteria. 

**Figure 2:**
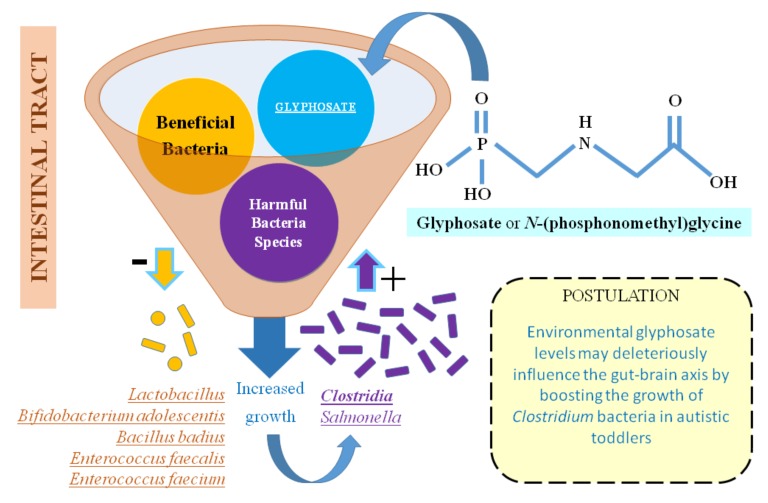
Glyphosate (GLY) may hypothetically contribute to ASD etiology by deleteriously influencing gut microbiome. Literature evidence suggests the link between Clostridium bacteria and developing and/or aggravating autism among children, highlighted by the present systematic review. Environmental exposure to GLY could contribute to such interplay by affecting the growth of normal gastrointestinal flora and therefore, allowing the growth of harmful bacteria species such as Clostridium, which are not sensitive to this pesticide.

All these taken together support our hypothesis (Figure 2) that exposure to environmental GLY may be an additional deleterious factor associated with the increase in *C. botulinum*-mediated diseases by suppressing the antagonistic effect of these healthy and beneficial gut bacteria in humans; and behind the interrelation between Clostridium bacteria colonization of the intestinal tract and autism etiology. the environmental contribution of GLY levels should be therefore further studied. Moreover, it should be considered that, besides GLY, there may be other toxins in the environment that Clostridium bacteria could be resistant to.

Finally, the identification of Clostridium bacteria as a potential etiological hallmark of ASD could have opened effective treatment possibilities for these patients, such as antimicrobial agents focused on the specific elimination of these microorganisms. To the best of our knowledge, the only study of any antimicrobial therapy in ASD patients (regressive-onset autism) was performed by Sandler and colleagues [46], who tested the short-term benefit from oral vancomycin treatment in 11 children with promising results.

## 5. Conclusions

The results of the present systematic review demonstrate an association between Clostridium bacteria colonization of the intestinal tract and ASD. In addition, we also postulate about how environmental GLY levels may deleteriously influence the gut-brain axis by boosting the growth of Clostridium bacteria in these patients.

## Figures and Tables

**Table 1:** Evidence for the link between Clostridium bacteria and autism spectrum conditions. Table summarizing the main results of nine studies included in the systematic review. The table is organized by year of publication.

TITLE	AUTHOR/YEAR	PMID	MATERIAL	N° PATIENTS/CONTROLS	CONCLUSIONS
Gastrointestinal microflora studies in late-onset autism	Finegold et al., 2002	12173102	Human (Stools)	13 ASD/8 controls	Children with autism had nine species of Clostridium not found in controls, with significant alterations in the upper and lower intestinal flora of children with late-onset autism
Real-time PCR quantitation of clostridia in feces of autistic children	Song et al., 2004	15528506	Human (Stools)	15 ASD/8 controls	Analysis of the real-time PCR data showed cell count differences between autistic and control children for *Clostridium bolteae*
Differences between the gut microflora of children with autistic spectrum disorders and that of healthy children	Parracho et al., 2005	16157555	Human (Stools)	58 ASD/22 controls (2 groups)	The fecal flora of autism spectrum disorder (ASD) patients contained a higher incidence of the *Clostridium histolyticum* group (Clostridium clusters I and II) of bacteria than that of healthy children
Fecal lactoferrin and *Clostridium* spp. in stools of autistic children	Martirosian et al., 2011	21167951	Human (Stools)	41 ASD/10 controls	Elevated level of fecal lactoferrin was demonstrated in 24.4% stools, all from boys (31.25%). No toxins were detected. *Clostridium* spp. was isolated with similar frequency from all samples. *Clostridium perfringens* were isolated significantly often from the autistic stools
Fecal microbiota and metabolome of children with autism and pervasive developmental disorder not otherwise specified	De Angelis et al., 2013	24130822	Human (Stools)	20 ASD/10 controls	The highest microbial diversity was found in ASD children. Based on 16S-rRNA and culture-dependent data, Faecalibacterium and Ruminococcus were present at the highest level in fecal samples of pervasive developmental disorder not otherwise specified (PDD-NOS) and healthy children. Caloramator, Sarcina and Clostridium genera were the highest in ASD children
Urinary 3-(3-Hydroxyphenyl)-3-hydroxypropionic Acid, 3-Hydroxyphenylacetic Acid, and 3-Hydroxyhippuric Acid Are Elevated in Children with Autism Spectrum Disorders	Xiong et al., 2016	27123458	Human (Urine)	62 ASD/62 controls	Measurement of these three compounds (aromatic metabolites in autism patients are presumably derived from overgrown Clostridium species in gut) are strong predictors of ASDs and support the potential clinical utility for identifying a subgroup of ASD subjects
Intestinal Dysbiosis and Yeast Isolation in Stool of Subjects with Autism Spectrum Disorders	Iovene et al., 2017	27655151	Human (Stools)	47 ASD/33 controls	Significant linear correlation was found between disease severity and calprotectin and *Clostridium* spp. presence in the stool of subjects with ASD
Analysis of the Duodenal Microbiome in Autistic Individuals: Association with Carbohydrate Digestion	Kushak et al., 2017	27811623	Human (Intestinal biopsies)	21 ASD/19 controls	A positive correlation was found between the abundance of Clostridium species and disaccharidase activity in autistic individuals
Detection of *Clostridium perfringens* toxin genes in the gut microbiota of autistic children	Finegold et al., 2017	28215985	Human (Stools)	33 ASD/13 controls	The author´s results indicate that autistic subjects with gastrointestinal disease harbor statistically significantly higher counts of *C. perfringens* and β2-toxin gene-producing *C. perfringens* in their gut when compared to control children. In addition, the incidence of β2-toxin gene-producing *C. perfringens* is also significantly higher in these autistic subjects
